# The Evolution of Spatial Omics Technologies Introduces A Novel Avenue for Lung Cancer Research

**DOI:** 10.1093/gpbjnl/qzag010

**Published:** 2026-01-30

**Authors:** Yue He (何越), Zifan Li (李紫凡), Wenxiang Wang (王文香), Xu Liu (刘旭), Shanshan Lu (卢珊珊), Jing Bai (白晶), Lin Weng (翁琳), Qingna Zhang (张庆娜), Jun Wang (王俊), Kezhong Chen (陈克终)

**Affiliations:** Department of Thoracic Surgery, Peking University People’s Hospital, Beijing 100044, China; Thoracic Oncology Institute, Peking University People’s Hospital, Beijing 100044, China; Research Unit of Intelligence Diagnosis and Treatment in Early Non-small Cell Lung Cancer, Chinese Academy of Medical Sciences, 2021RU002, Peking University People’s Hospital, Beijing 100044, China; Institute of Advanced Clinical Medicine, Peking University, Beijing 100191, China; Department of Thoracic Surgery, Peking University People’s Hospital, Beijing 100044, China; Thoracic Oncology Institute, Peking University People’s Hospital, Beijing 100044, China; Research Unit of Intelligence Diagnosis and Treatment in Early Non-small Cell Lung Cancer, Chinese Academy of Medical Sciences, 2021RU002, Peking University People’s Hospital, Beijing 100044, China; Institute of Advanced Clinical Medicine, Peking University, Beijing 100191, China; Department of Thoracic Surgery, Peking University People’s Hospital, Beijing 100044, China; Thoracic Oncology Institute, Peking University People’s Hospital, Beijing 100044, China; Research Unit of Intelligence Diagnosis and Treatment in Early Non-small Cell Lung Cancer, Chinese Academy of Medical Sciences, 2021RU002, Peking University People’s Hospital, Beijing 100044, China; Institute of Advanced Clinical Medicine, Peking University, Beijing 100191, China; Department of Thoracic Surgery, Peking University People’s Hospital, Beijing 100044, China; Thoracic Oncology Institute, Peking University People’s Hospital, Beijing 100044, China; Research Unit of Intelligence Diagnosis and Treatment in Early Non-small Cell Lung Cancer, Chinese Academy of Medical Sciences, 2021RU002, Peking University People’s Hospital, Beijing 100044, China; Institute of Advanced Clinical Medicine, Peking University, Beijing 100191, China; Department of Pathology, Peking University People’s Hospital, Beijing 100044, China; College of Future Technology, Peking University, Beijing 100871, China; Geneplus-Beijing Institute, Beijing 102206, China; Department of Thoracic Surgery, Peking University People’s Hospital, Beijing 100044, China; Thoracic Oncology Institute, Peking University People’s Hospital, Beijing 100044, China; Research Unit of Intelligence Diagnosis and Treatment in Early Non-small Cell Lung Cancer, Chinese Academy of Medical Sciences, 2021RU002, Peking University People’s Hospital, Beijing 100044, China; Institute of Advanced Clinical Medicine, Peking University, Beijing 100191, China; Department of Thoracic Surgery, Peking University People’s Hospital, Beijing 100044, China; Department of Thoracic Surgery, Peking University People’s Hospital, Beijing 100044, China; Thoracic Oncology Institute, Peking University People’s Hospital, Beijing 100044, China; Research Unit of Intelligence Diagnosis and Treatment in Early Non-small Cell Lung Cancer, Chinese Academy of Medical Sciences, 2021RU002, Peking University People’s Hospital, Beijing 100044, China; Institute of Advanced Clinical Medicine, Peking University, Beijing 100191, China; Department of Thoracic Surgery, Peking University People’s Hospital, Beijing 100044, China; Thoracic Oncology Institute, Peking University People’s Hospital, Beijing 100044, China; Research Unit of Intelligence Diagnosis and Treatment in Early Non-small Cell Lung Cancer, Chinese Academy of Medical Sciences, 2021RU002, Peking University People’s Hospital, Beijing 100044, China; Institute of Advanced Clinical Medicine, Peking University, Beijing 100191, China

**Keywords:** Spatial proteomics, Spatial transcriptomics, Lung cancer, Tumor heterogeneity, Tumor microenvironment

## Abstract

Lung cancer is a highly malignant disease, posing a significant threat to global health. The presence of tumor heterogeneity results in substantial variations in prognosis and therapeutic response among patients. Advances in bulk RNA sequencing and single-cell RNA sequencing have facilitated the identification of driver gene mutations and the exploration of cellular diversity within tumors. However, tumors are complex ecosystems comprising both tumor cells and their microenvironment, where interactions among different cell types give rise to specific functional and structural units that collectively drive tumorigenesis and progression. The emergence of spatial omics technologies has allowed for the analysis of tumor ecosystems, providing unprecedented insights into tumor heterogeneity. This review presents updates on spatial omics technologies and data analysis algorithms, discusses current technical limitations, and explores potential future developments. Furthermore, we summarize the latest applications of spatial omics in elucidating lung cancer heterogeneity, investigating mechanisms of lung cancer progression and drug resistance, and identifying novel biomarkers. Drawing from these insights, we propose strategies for integrating spatial omics into lung cancer research, offering new perspectives for precision medicine.

## Graphical abstract

**Figure:**
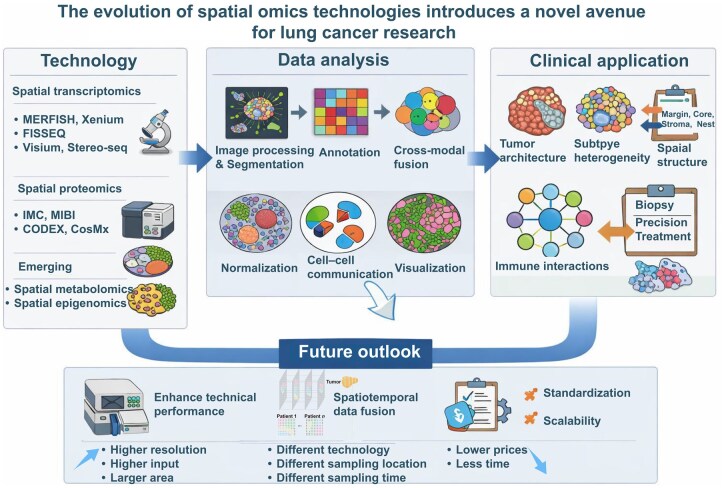

## Introduction

Lung cancer is one of the major issues threatening public health [1,2]. Because of its apparent heterogeneity, patients exhibit varying sensitivities to treatment, resulting in some with poor treatment responses and dismal prognosis [1,2]. Tumor heterogeneity can be divided into inter-tumor heterogeneity and intra-tumor heterogeneity [3]. Inter-tumor heterogeneity reflects the cumulative effects of genetic and epigenetic aberrations acquired during the long course of each tumor, contributing to the variability among individuals with tumors of the same histologic type [4]. The development of batch sequencing technologies enables us to identify the driver genes of lung cancer, thereby unveiling the mystery of inter-tumor heterogeneity and allowing us to design more personalized targeted treatment plans for patients carrying driver genes [5]. Intra-tumor heterogeneity is driven by genetic, epigenetic, and microenvironmental factors, reflecting the existence of multiple cell subtypes in the same tumor [6–8]. The advent of single-cell RNA sequencing (scRNA-seq) provides unprecedented resolution to delineate the cellular heterogeneity of tumors and tumor microenvironments (TMEs) [9,10]. However, tumors are structurally complex ecosystems [11]. Cells do not act in isolation; they communicate with each other, collectively influencing tumor behavior. Consequently, it is essential to uncover the spatial distribution patterns of various cell subtypes, a task unattainable solely through single-cell sequencing.

Spatial omics technologies enable the detection of multiple molecular layers — including transcripts, proteins, metabolites, and epigenetic modifications — while simultaneously obtaining their spatial positions within tissues. These technologies have been extensively utilized in lung cancer research in recent years, driven by continued advances. With these technologies, the spatial architecture of tumor tissues can be mapped, enabling higher-dimensional analysis of tumor heterogeneity and a deeper understanding of the lung cancer ecosystem. The ultimate goal is to predict clinical outcomes and provide timely, individualized interventions based on spatial indicators.

## Advances in spatial omics technologies

### Spatial transcriptomics technologies

**Figure 1:**
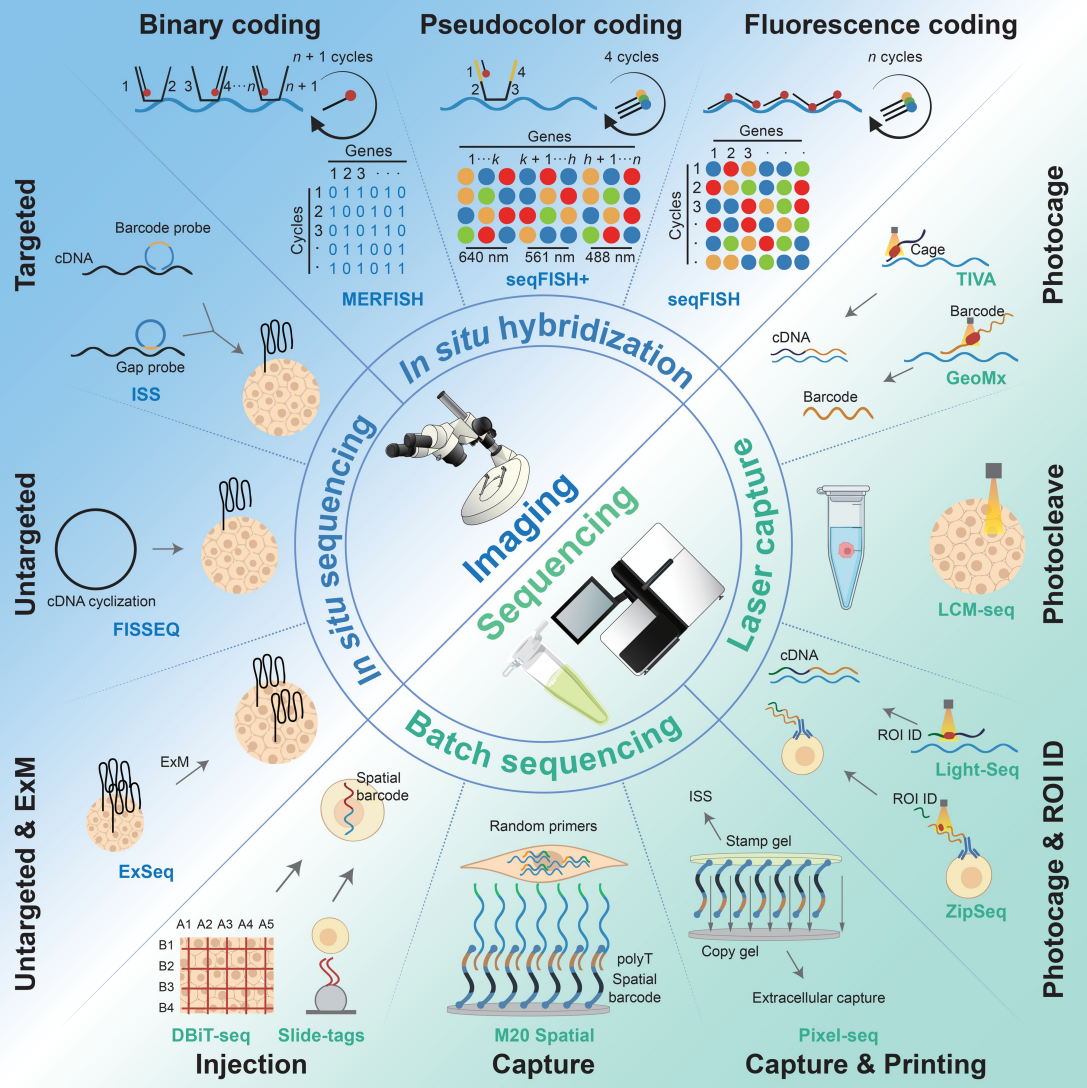
Overview of ST technologies ST technologies can be categorized into four types. ISH- and ISS-based technologies primarily rely on imaging output, whereas bulk sequencing and laser capture-based technologies rely on sequencing output. Compared with sequencing technologies, imaging technologies have high spatial resolution but relatively low throughput. ST, spatial transcriptomics; MERFISH, multiplexed error-robust fluorescence *in situ* hybridization; seqFISH, sequential fluorescence *in situ* hybridization; TIVA, transcriptome *in vivo* analysis; LCM-seq, laser capture microdissection sequencing; DBiT-seq, deterministic barcoding in tissue sequencing; ExSeq, expansion sequencing; FISSEQ, fluorescent *in situ* sequencing; ExM, expansion microscopy.

Spatial transcriptomics (ST) typically refers to the measurement of the mRNA expression of a large number of genes in the spatial context of tissues [12]. At its inception, ST technologies were immature and exhibited drawbacks such as poor sensitivity, low throughput, high cost, low resolution, or difficulty in defining boundaries between individual cells. In recent years, a multitude of ST technologies have emerged to address the aforementioned issues (**Figure 1**; **Table 1**).

**Table 1:** Comparison of ST technologies

Type	Name	Sample	Resolution	Throughput	Capture area	Characteristic	No. of lung cancer publications	Technical ref.	Advantage	Disadvantage
ISH-based technologies	seqFISH	FF	Subcellular	–	Slides	Using sequential fluorescent barcoding	0	[13,14]	High resolution High sensitivity	Unique probe design is required High experimental equipment requirements
	Xenium	FFPE, FF	Subcellular	∼ 5000	Slides (10.5 × 22.5 cm^2^)	Using sequential fluorescent barcoding Preserved tissue integrity aligns with H&E sections commercialization	1	[15]		
	CosMx	FFPE, FF	∼ 50 nm	∼ 6000	Slides (cm^2^)	Using sequential fluorescent barcoding Commercialization	3	[16]		
	seqFISH+	FF	Subcellular	∼ 10,000	Slides (mm^2^)	Fluorescence coding using 60 pseudocolorchannels	1	[17]		
	MERFISH	FF	Subcellular	∼ 500	Slides (cm^2^)	Binary coding Error robustness Commercialization	3	[18]		
	osmFISH	FF	Subcellular	<100	Slides (mm^2^)	Nonbarcoded Wide dynamic range	0	[19]		
	clampFISH	FF	Subcellular	∼ 3	Slides	Signal amplification by iterative hybridization	0	[20]		
	Scrinshot	FFPE, FF	Cellular	∼ 30	Slides	Signal amplification by RCA	0	[21]		
Targeted ISS-based technologies	ISS	FF	Subcellular	∼ 250	Slides (mm^2^)	Gap-targeted probes and barcode-targeted probes	0	[22]	Signal amplification by RCA High resolution	Low throughput Unique probe design is required
	HybISS	FF	Subcellular	∼ 120	Slides (mm^2^)	Increased SNR using SBH	0	[23]		
	BaristaSeq	–	–	–	–	Using Phusion DNA polymerase without strand displacement activity Increased SNR using SBS	0	[24]		
	STARmap	FF	Subcellular	∼ 1000	Slides (mm^2^)	Improved spatial accuracy by tissue expansion Improved specificity by two-probe hybridization	0	[25]		
Untargeted ISS-based technologies	FISSEQ	FFPE, FF	Subcellular	Transcriptome-wide	Slides (mm^2^)	Cyclization of cDNA	0	[26,27]	High throughput and high resolution are expected	Low sensitivity at present High cost
	ExSeq	FF	Subcellular	Transcriptome-wide	Slides (mm^2^)	Cyclization of cDNA Improved spatial accuracy by tissue expansion	0	[28]		
Laser capture-based technologies	LCM-seq	FFPE, FF	Cellular	Transcriptome-wide	ROIs	The combination of LCM and Smart-seq2	0	[29]	High throughput	Data are often aggregations of ROIs containing multiple cells
	TIVA	Live cells	Cellular	Transcriptome-wide	ROIs	Photoactivatable mRNA capture molecules	0	[30]		
	PSS	FF	Subcellular	Transcriptome-wide	ROIs	Tn5 transposons with photocleavable adapters	0	[31]		
	GeoMx	FFPE, FF	10 μm	Transcriptome-wide	ROIs	Oligo-labeled probes with light-cleavable linkers Commercialization	17	[32]		
	ZipSeq	Live tissues	Cellular	Transcriptome-wide	ROIs	NPOM caged O1 Different ROIs distinguished by zipcodes	0	[33]		
	Light-Seq	FF	Cellular	Transcriptome-wide	ROIs	Light-controlled DNA barcodes attachment No sample damage Different ROIs distinguished by region ID	0	[34]		
Batch sequencing-based technologies	10x Visium	FFPE, FF	100 µm	Transcriptome-wide	Slides (6.5 × 6.5 mm^2^)	Simple process and high usability Commercialization	6	[35,36]	Unbiased full transcriptome analysis of whole slides Standardized process	High cost Difficulty in dividing cell boundaries (except Slide-tags)
	Visium HD	FFPE, FF	2 µm	Transcriptome-wide	Slides (6.5 × 6.5 mm^2^)	Simple process and high usability Commercialization	1	[37]		
	Slide-seq	FF	10 µm	Transcriptome-wide	Slides (mm^2^)	Bead array	0	[38,39]		
	HDST	FF	2 µm	Transcriptome-wide	Slides (5.7 × 2.4 mm^2^)	Bead array	0	[40]		
	Seq-Scope	FF	0.6 μm	Transcriptome-wide	Slides (mm^2^)	Probe cluster	0	[41]		
	Stereo-seq	FF	0.5 μm	Transcriptome-wide	Slides (13.2 × 13.2 cm^2^)	DNA nanoball Commercialization	1	[42]		
	Pixel seq	FF	1 µm	Transcriptome-wide	Slides (9.0 × 35 mm^2^)	Polony gel stamping	0	[43]		
	M20 Spatial	FFPE, FF	15 µm	Transcriptome-wide	Slides (mm^2^)	Full-length transcriptome sequencing Commercialization	0	[44]		
	DBiT-seq	FF	10 µm	Transcriptome-wide	Slides (mm^2^)	Spatial barcodes delivery	0	[45]		
	Patho-DBiT-seq	FFPE, FF	10 µm	Transcriptome-wide	Slides (mm^2^)	High sensitivity in FFPE samples	0	[46]		
	Slide-tags	FF	20 µm	Transcriptome-wide	Slides (mm^2^)	Spatial barcodes delivery	0	[47]		
	Open-ST	FF	0.6 μm	Transcriptome-wide	Slides (3.0 × 4.0 mm^2^)	Relatively low cost 3D tissue reconstruction	0	[48]		

*Note*: ST, spatial transcriptomics; ISH, *in situ* hybridization; ISS, *in situ* sequencing; FFPE, formalin-fixed paraffin-embedding; FF, fresh frozen; ROIs, regions of interest; RCA, rolling circle amplification; LCM, laser capture microdissection; SBH, sequence-by-hybridization; SBS, sequencing by synthesis; SNR, signal-to-noise ratio.

#### *In situ* hybridization-based technologies

*In situ* hybridization (ISH)-based technologies primarily employ fluorescent probes to hybridize with target transcripts in the native tissue environment for visualization. The challenge lies in utilizing a limited number of fluorophores while still achieving high-throughput determination. Sequential fluorescence *in situ* hybridization (seqFISH) pioneers the use of fluorescence coding to significantly improve the detection throughput [13,14]. CosMx further pushes fluorescent coding toward commercialization [16]. By encoding barcodes with pseudocolor, a revised version of seqFISH, known as seqFISH+, enhances throughput, efficiency, and error reduction [17]. Another method, multiplexed error-robust FISH (MERFISH), transforms sequential barcodes derived from the color order into binary barcodes based on fluorescence presence or absence [49]. MERFISH has been commercialized for its ability to simultaneously detect 500 genes at subcellular resolution [18]. Notably, Xenium employs rolling circle amplification (RCA) to amplify the readout probes via the bridge probes, thereby enhancing fluorescent signals [15]. These ISH-based methods have demonstrated robust performance on formalin-fixed paraffin-embedded (FFPE) samples, which represent the predominant clinical specimen resource. Their short probe designs effectively target fragmented RNA in FFPE tissues, achieving good detection sensitivity at subcellular resolution [50,51].

#### *In situ* sequencing-based technologies

*In situ* sequencing (ISS)-based technologies capture transcripts in the native tissue environment and then amplify the signal through RCA to generate DNA nanospheres for sequencing. The initial ISS technology involves the design of two types of targeted probes: gap-targeted and barcode-targeted probes. By sequencing specific gap sequences and DNA barcodes *in situ*, the RNA type can be determined [22]. Due to the use of targeted probes, ISS is primarily suitable for analyzing selected genes of interest. In contrast, untargeted technologies allow for whole transcriptome analysis. Fluorescent *in situ* sequencing (FISSEQ) directly immobilizes and circularizes cDNA *in situ* for subsequent sequencing-by-ligation (SOLiD) sequencing, thereby overcoming the throughput limitations of targeted probes [26,27]. However, high throughput also brings higher amplicon density, which leads to lower sequencing depth and sensitivity. Another technology, expansion sequencing (ExSeq), partially addresses the above problem of molecular crowding by combining expansion microscopy (ExM) with FISSEQ. At the same time, the *in situ* sequencing results will be matched with the subsequent *ex situ* next-generation sequencing, which is expected to achieve high spatial accuracy of long transcript sequencing [28].

#### Laser capture-based technologies

The development of laser capture microdissection (LCM) enables tissues to be dissected at the micron resolution to obtain regions of interest (ROIs) [52]. Coupling LCM with RNA sequencing directly links transcriptomic data to their precise spatial context. LCM-seq serves as a successful example, combining LCM with Smart-seq2 to select trace cells or even single cells in tissues for transcriptome analysis [29]. However, excision may lead to sample contamination by neighboring cells and can also be time-consuming when multiple ROIs are required. In recent years, laser capture-based technologies have evolved from physical cutting to photolysis of photocaging groups. These technologies first keep the probe in an inactive state via photocaging groups; upon laser irradiation of the ROI, the photocaging groups are photocleaved, thereby obtaining transcriptome information at selected spatial locations. For example, transcriptome *in vivo* analysis (TIVA) blocks probe binding to RNA by attaching a photocaging group to the polyU tail of the probe [30]. GeoMx utilizes barcode probes with photocleavable adapters and subsequently irradiates the ROIs with a laser to obtain specific barcodes for sequencing [32]. These technologies have achieved subcellular resolution but are less efficient because they require sequential extraction of transcript information for each ROI. To address this issue, some technologies label ROIs with spatial barcodes, enabling the distinction of transcripts from different ROIs. In Light-Seq, spatial barcodes containing the ultrafast photo-cross-linker 3-cyanovinylcarbazole nucleoside (CNVK) are photo-cross-linked *in situ* to cDNA, while ZipSeq directly designs probes containing spatial barcodes [33,34]. With the assistance of spatial barcodes, transcripts from different ROIs can be sequenced simultaneously.

#### Batch sequencing-based technologies

Batch sequencing-based technologies represent the most rapidly advancing and widely commercialized approaches within the field of ST. One of the earliest batch sequencing-based technologies is 10x Visium, which deposits ordered capture probe arrays onto a slide using microarray printing [35,36]. Its second generation, Visium HD, achieves single-cell resolution [37]. Furthermore, this company has introduced spatial full-length transcriptome sequencing technology (M20 Spatial), enabling in-depth exploration of gene fusions and alternative splicing at the spatial level [44]. Visium has established dedicated FFPE workflows with optimized protocols, though gene detection rates remain lower compared to fresh-frozen samples [53].

Since spatial resolution is influenced by probe density on the chip, it is necessary to increase the probe density within a given range to enhance the spatial resolution. Slide-seq and high-definition ST (HDST) initially package the probes onto beads, which are then mounted onto a glass slide, thereby increasing the resolution to approximately 10 μm and 2 μm, respectively [38–40]. Seq-Scope performs solid-phase amplification of the probe to generate probe clusters, which further improves its resolution to approximately 0.6 μm [41]. Stereo-seq produces DNA nanoballs (DNB) through the rolling circle amplification of the probe, enabling a broader spatial barcode pool and achieving approximately 0.5 μm resolution [42]. However, these technologies increase technical complexity due to the requirement for sequencing spatial barcodes. Pixel-seq solves this problem through the utilization of polony gel stamping [43]. Recently, Open-ST (open spatial transcriptomics) has emerged as a cost-effective and accessible platform that combines next-generation sequencing (NGS)-based spatial barcoding with standard histology workflows, achieving subcellular resolution while maintaining compatibility with existing laboratory infrastructure [48,54].

The above technologies use probe capture chips to capture RNA from permeabilized tissues. A major challenge is that lateral diffusion of RNA and differences in cell permeability levels may lead to increased sequencing errors [45]. Other technologies, such as DBiT-seq and Slide-tags, effectively avoid this problem by injecting spatial barcodes into cells [45,55]. Building upon DBiT-seq, Patho-DBiT-seq has been specifically optimized for FFPE tissue sections through enhanced permeabilization and enzymatic pretreatment, demonstrating promising results for clinical pathology applications [45,56]. At present, spatial barcode delivery-based technologies are still in their infancy, and further methodological innovation and optimization are needed.

### Spatial proteomics technologies

**Figure 2:**
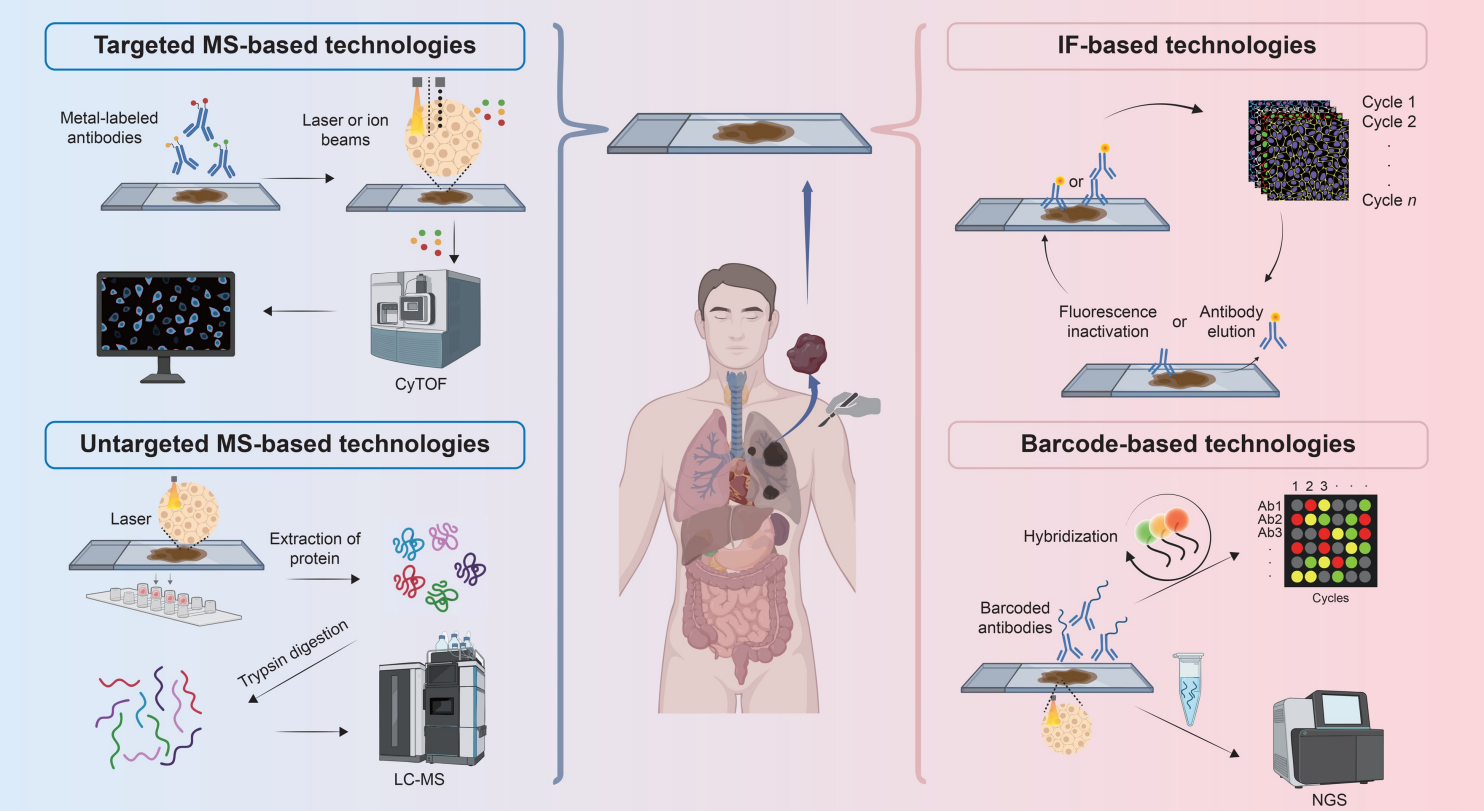
Overview of SP technologies SP technologies can be categorized into four types. Compared with the other three technologies, untargeted MS-based technologies offer significantly higher detection throughput. However, due to the difficulty in accurately cutting single cells, these technologies are currently used less frequently. SP, spatial proteomics; LC-MS, liquid chromatography mass spectrometry; NGS, next-generation sequencing.

ST technologies undeniably provide us with rich information, but the translation of mRNAs into their homologous proteins is highly regulated and nonlinear [57]. RNA expression cannot directly predict protein expression. Therefore, spatial proteomics (SP) technologies may more accurately reflect specific cellular functions and states (**Figure 2**; **Table 2**).

**Table 2:** Comparison of SP technologies

Type	Name	Sample	Resolution	Throughput	Capture area	Characteristic	No. of lung cancer publications	Technical ref.	Advantage	Disadvantage
Targeted MS-based technology	IMC	FFPE, FF	1 μm	∼ 40	Slides (mm^2^)	Using metal-coupled antibodies Commercialization	37	[58]	No sample autofluorescence Wide dynamic range	Slow detection speed Unique antibody design is required
	MIBI	FFPE, FF	250 nm	∼ 40	Slides (mm^2^)	Using metal-coupled antibodies Commercialization	5	[59]		
Untargeted MS-based technologies	nanoPOTS	FFPE, FF	∼ 10 cells	1500–3000	ROIs	Compatible with multiple workflows	0	[60,61]	Untargeted detection High throughput	Low resolution
	DVP	FFPE, FF	200 nm	∼ 5000	ROIs	Image analysis using AI	0	[62]		
	DISCO-MS	FF	200 μm	∼ 1000	ROIs	3D imaging of the entire specimen	0	[63]		
IF-based technologies	CycIF	FFPE, FF	110 nm	∼ 60	Slides	Pre-staining to reduce auto-fluorescence Using conventional microscopes	3	[64]	Low requirements for experimental equipment High spatial resolution	Sample autofluorescence Sample destruction and SNR reduction due to repeated loops
	MxIF	FFPE, FF	Subcellular	∼ 61	Slides	Mild fluorescence inactivating reagents	0	[65]		
	4i	FFPE, FF	165 nm	∼ 40	Slides	The imaging buffer can avoid free radical mediated antibody cross-linking with the sample	0	[66]		
	MICS	FFPE, FF	Subcellular	∼ 300	Slides	New image processing algorithms (MACS iQ View)	0	[67]		
Barcode-based technologies	CODEX	FFPE, FF	250 nm	∼ 40	Slides (cm^2^)	Using sequential fluorescent barcoding Commercialization	6	[68,69]	No sample autofluorescence Standardized experimental procedures	Unique antibody design is required
	CosMx	FFPE, FF	∼50 nm	∼ 100	Slides (cm^2^)	Using sequential fluorescent barcoding Commercialization	3	[16]		
	DEI	FFPE, FF	Subcellular	∼ 8	Slides	Based on DNA PAINT Fast imaging speed	0	[70,71]		
	Immuno-SABER	FFPE, FF	Subcellular	∼ 10	Slides (mm^2^)	Signal amplification by PER High sensitivity Commercialization	0	[72,73]		
	GeoMx	FFPE, FF	10 μm	∼ 570	ROIs	Oligo-labeled antibodies with light-cleavable linkers Commercialization	17	[32]		

*Note*: SP, spatial proteomics; MS, mass spectrometry; IF, immunofluorescence; FFPE, formalin-fixed paraffin-embedding; FF, fresh frozen; PER, primer exchange reaction.

#### Targeted mass cytometry-based technologies

Imaging mass cytometry (IMC) and Multiplexed ion beam imaging (MIBI) are two commonly used targeted mass spectrometry-based technologies for spatial proteomic analysis [58,59]. In IMC, antibodies are conjugated with an exclusive rare-earth metal isotope of specified atomic mass. Subsequently, following targeted laser ablation, the metal isotopes are transported to the CyTOF mass spectrometer for analysis in aerosol form, allowing for characterization of different antigens based on the mass of the isotopes. The distinction between MIBI and IMC lies in how they handle metal isotopes. MIBI employs ion beams to impact metal particles, generating metal ions that are subsequently released and captured for analysis by a mass spectrometer. Both methods offer precise spatial analysis of antigen localization at subcellular resolution.

#### Untargeted MS-based technologies

Untargeted MS-based technologies first cleave the regions of interest (ROIs) and then conduct high-depth proteomic analysis using ultra-sensitive mass spectrometry. For instance, the nanodroplet processing platform has developed a chip called nanoPOTS, which allows the transfer of ROIs obtained by LCM onto the chip for analysis using liquid chromatography mass spectrometry (LC-MS) at a depth of up to 2000 proteins [60,61]. To enhance resolution to single cells or even subcellular levels, scientists have combined artificial intelligence with mass spectrometry, resulting in the development of a new technology called Deep Visual Proteomics (DVP) [62]. DVP employs deep learning and machine learning (ML) to delineate cell boundaries and nuclei in high-resolution sample sections. This enables subsequent microdissection at the single-cell or subcellular level for mass spectrometry analysis. In comparison to targeted MS-based technologies, untargeted MS-based technologies achieve significantly higher detection throughput but lower spatial resolution.

#### Immunofluorescence-based technologies

The development of immunofluorescence (IF)-based technologies is relatively early, with numerous technologies emerging. However, the increase in cycling causes a decrease in signal-to-noise ratio (SNR) and an increase in sample destruction. To overcome these problems, MxIF and CellScape designed a gentle fluorescence inactivation process to preserve sample integrity after multiple cycles [65,74]. Iterative indirect immunofluorescence imaging (4i) employs an imaging buffer containing a radical scavenger and an acceptor for free radical-induced photo-cross-linking, which can prevent free radical-mediated antibody cross-linking to the sample, thereby improving antibody elution rates [66].

#### Barcode-based technologies

The most commonly used barcode-based technologies are codetection by indexing (CODEX) and CosMx, which combine antibodies with DNA barcodes, attach fluorescent probes to the DNA barcodes, and enable batch antibody visualization through iterative cycles [16,68,69]. Building on this, DNA Exchange Imaging (DEI) further simplifies the experimental process by utilizing DNA-based point accumulation for imaging in nanoscale topography (DNA-PAINT), which enables the spontaneous binding and detachment of fluorescent probes from DNA barcodes [70,71] Immune-SABER amplifies the fluorescent probe attachment sites through the primer exchange reaction (PER), significantly increasing fluorescence intensity and thereby enhancing sensitivity [72,73]. In addition, there are also technologies that extend beyond this principle to perform “sequencing” in practice. GeoMx attaches oligonucleotide probes with photocleavable linkers to the antibody, enabling probes to be collected for sequencing by laser irradiation of the ROIs [32]. GeoMx offers the advantages of simple operation and high automation. However, it does not provide proteomic information for each cell in a tissue section.

## Emerging spatial omics technologies

Beyond ST and SP, spatial metabolomics and spatial epigenomics are rapidly emerging as complementary dimensions for comprehensive tissue characterization.

Spatial metabolomics technologies enable visualization and quantification of metabolite distributions within tissues. Mass spectrometry imaging (MSI)-based approaches have advanced significantly, with recent developments improving sensitivity and spatial resolution [75]. Emerging platforms combine MSI with capillary electrophoresis for enhanced molecular characterization [76]. Novel integration methods now enable simultaneous spatial metabolomics and transcriptomics analyses on the same tissue sections, revealing metabolite–transcript correlations in cancer tissues [77].

Spatial epigenomics technologies map chromatin states, histone modifications, and DNA methylation in a spatial context. Spatial-CUT&Tag enables genome-wide profiling of histone modifications at cellular resolution in tissue sections [78,79]. Recent protocols for spatial-ATAC-RNA-seq and spatial-CUT&Tag-RNA-seq allow joint profiling of epigenome and transcriptome on the same tissue sections [78,80]. SPACE-seq developed in 2025, enables simultaneous analysis of gene expression, chromatin accessibility, and mitochondrial DNA mutations using standard ST platforms [81]. These approaches provide insights into gene regulatory mechanisms underlying spatial heterogeneity.

Although currently less mature than ST and SP technologies, spatial metabolomics and epigenomics are experiencing rapid growth and hold great promise for multi-omics integration, offering a more holistic view of disease mechanisms in lung cancer research.

## Recent advances in ST analysis methods

**Figure 3:**
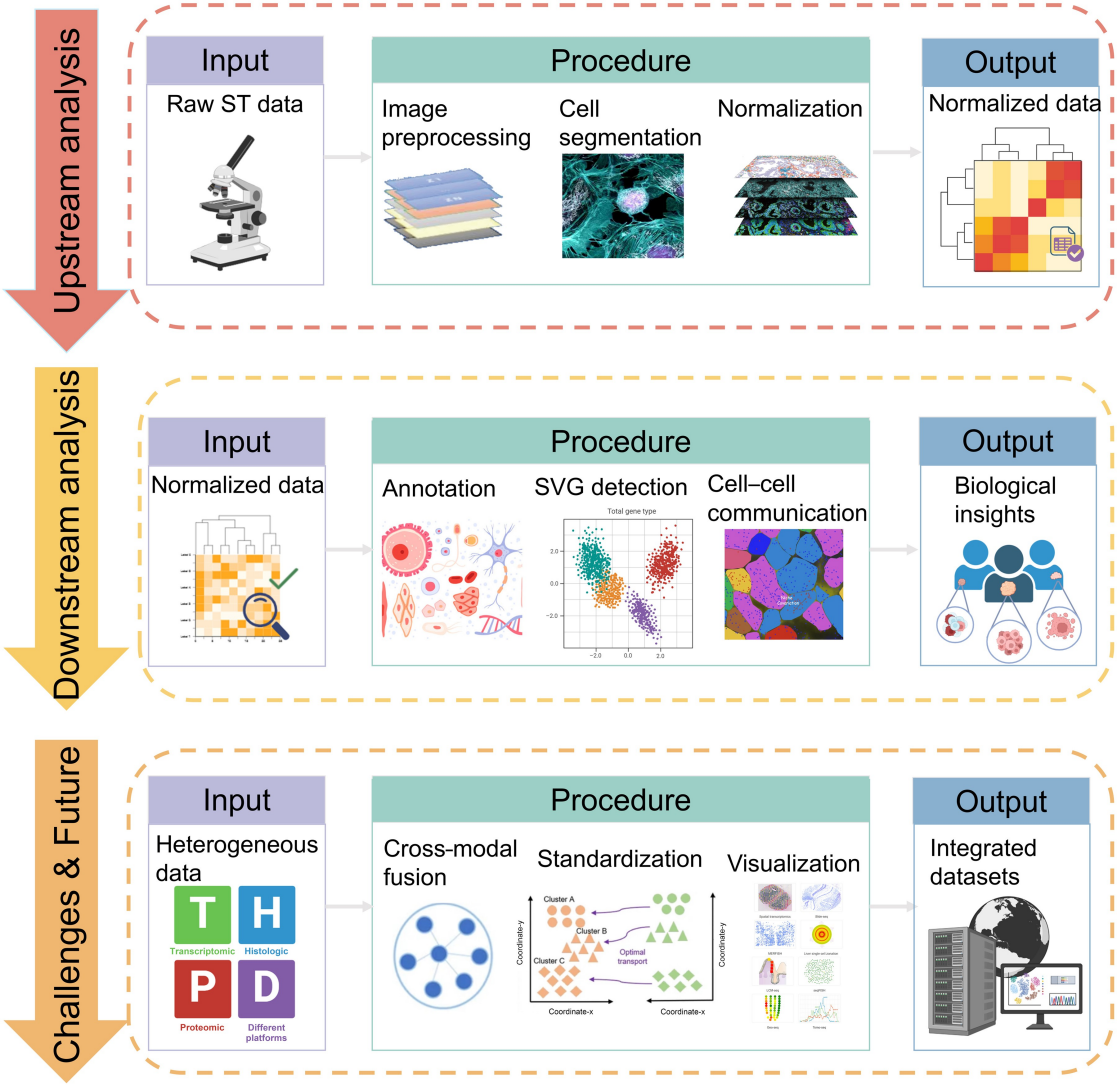
Schematic flow pattern for routine analysis of spatial omics The workflow is divided into three main modules: upstream analysis, downstream analysis, challenges & future. Each module represents a critical stage in the analysis pipeline, with arrows indicating the flow of data and analytical steps. SVG, spatially variable gene.

Spatial omics, as an emerging field, has spurred the development of numerous complementary analytical packages and methodologies. In this section, we provide an overview of the latest methodologies encompassing cell segmentation, data preprocessing, identification of spatially variable genes, clustering, and subsequent analyses, including molecular crosstalk and pseudotime analysis (**Figure 3**).

### Upstream analysis: image processing and data preprocessing

Upstream analysis typically encompasses image preprocessing, specimen optimization, quality control, and normalization, transforming raw biological samples into normalized data suitable for subsequent analysis.

For image-based ST data such as MERFISH and seqFISH, processing primarily involves image registration, transcript spots identification, and cell segmentation [12]. Background correction of the image is essential to eliminate non-specific background signals. A variety of methods, such as DeepBlink [82], gcrma [83], and affy [84], are now available for automated processing of images with varying background brightness levels and spot qualities. They identify the location and expression of RNA in the image, which, for some techniques, may be the protein count, and generate an associated location index matrix and a gene expression matrix. When the analyzed object is too large to be captured in a single section, it is common to use multiple slices, by adding Z-stack coordinates and constructing a spatial distribution matrix [85,86]. Analyzing 3D data at the 2D level is bound to produce artifacts and misalignments. Researchers have mitigated such batch effects by developing new models for integrating spatial coordinate system localization and hierarchical feature extraction [87,88]. The role of artificial intelligence (AI) and deep learning in spatial omics analysis has become increasingly pivotal, fundamentally transforming how we process and interpret ST data. Deep learning models have demonstrated superior performance in cell segmentation tasks. Packages such as ilastik, DeepCell-TF, and Cellpose employ convolutional neural networks (CNNs) to achieve automated and accurate cell boundary detection [89,90]. These AI-driven approaches significantly outperform traditional image processing methods, particularly in handling complex tissue architectures and overlapping cellular structures [16,91]. When selecting among these tools, researchers should consider specific trade-offs: ilastik and DeepCell-TF offer superior accuracy for complex cellular morphologies but require substantial manual training and computational resources, while Cellpose provides an optimal balance between accuracy and efficiency for routine applications with moderate tissue complexity [90,92]. Recent benchmarking studies have revealed that method performance is highly dependent on tissue characteristics and imaging quality, emphasizing the importance of matching computational tools to specific experimental contexts [92,93]. Beyond segmentation, CNNs excel at processing ST images for tasks including automated tissue structure recognition, cell type classification, and spatial pattern detection with unprecedented accuracy. Graph neural networks (GNNs) have emerged as powerful tools for modeling cell–cell interactions by representing tissue architecture as graph structures, where nodes represent cells and edges capture spatial relationships [94,95]. Transfer learning approaches have proven particularly valuable in spatial omics, allowing adaptation of pre-trained models to new datasets, thereby reducing computational requirements and improving performance even with limited sample sizes — a common constraint in ST studies [96].

For sequencing-based ST data, such as 10x Visium, reads are obtained from distinct spatial spots. The overall analysis process is similar to that for single-cell transcriptomes. The difficulty lies in the accurate mapping of spot transcripts to spatial coordinates [97], a challenge that can be partially controlled through tissue optimization and quality control.

The concept of normalization originated from scRNA-seq to account for mRNA capture differences between dissociated cells and is equally applicable to ST. Inconsistent mRNA capture at different locations in the same section often requires normalization to unify the data and facilitate downstream analyses. Commonly used analysis packages, including Scanpy, Cell Ranger, Squidpy, Giotto, and Seurat, are compatible with this step through methods such as SCTransform, stLearn, and others [98–100].

### Downstream analysis: advanced computational analysis methods

Downstream analyses usually refer to analyses carried out on data after preliminary processing for various research purposes.

Since simple annotation of spatial spots may not reach single-cell resolution, cell-type determination and cellular composition estimation are often achieved by enrichment or deconvolution — for example, by assessing the enrichment of cell-specific expressed gene markers within each spot for cellular annotation. It is usually a qualitative process that indicates whether a certain cell is present within this spatial unit. Examples include the MIA method, Seurat’s own function AddModuleScore, and Seurat’s own coanalysis methods FindTransferAnchors and TransferData [101–103]. Deconvolution methods aim to quantitatively analyse the percentage of different cell types in each spatial spot. Negative binomial model and non-negative least squares (NNLS) are common principles of these methods, which map spatial spots containing unknown cellular mixture to annotated cellular atlases, such as SPOTlight, Stereoscope, spatialDWLS, and cell2location [103–106]. Comparative evaluations of these methods reveal distinct strengths suited to different analytical scenarios [107,108]. SPOTlight demonstrates superior computational efficiency and scalability, making it ideal for large-scale datasets, though it may show reduced accuracy in tissues with transcriptionally similar cell types. Stereoscope excels in resolving complex cellular compositions with overlapping signatures, particularly in heterogeneous tumor microenvironments, at the cost of increased computational time. Cell2location provides the most accurate estimates for rare cell populations through its probabilistic framework, but requires high-quality reference data and substantial computational resources. RCTD offers robust performance across diverse tissue types and maintains consistent accuracy with varying spot sizes, representing a reliable general-purpose option. RCTD calculates these proportions by weighting and summing the mean standardized expression values of known cell types from single-cell data by an unknown proportion, assuming that the spot expression count values conform to a Poisson distribution [109]. CellTrek, unlike deconvolution methods such as SPOTlight and RCTD, is characterized by integrating single-cell transcriptome data with ST data through co-embedding a newly created object [110]. Tangram represents a breakthrough in deep learning-based spatial deconvolution, directly learning correspondences between scRNA-seq and ST data through neural networks and estimating missing gene counts via soft assignment [111]. While Tangram achieves exceptional accuracy in spatial mapping and cross-platform integration, it demands substantial computational resources and longer runtime, which may limit its use in rapid exploratory analyses [107,112]. This exemplifies how AI methods can address fundamental challenges in ST by leveraging complex pattern recognition capabilities.

Spatially variable genes (SVGs) are genes with specific spatial expression patterns in tissues. Analysis algorithms include SpaGCN, which integrates gene expression using graph convolutional networks [113]; SPARK and GPcounts, which use statistical methods such as generalized linear models and non-parametric modelling [114]; SpatialDE, which is based on Gaussian process regression [115]; and Markvario and Moran’s I, which are based on quantifying the distance between two points [103]. The selection of appropriate SVG detection methods should be guided by dataset characteristics and analytical objectives. For large-scale datasets (> 10,000 spots), SPARK-X and SOMDE offer superior computational efficiency while maintaining high sensitivity. SpaGCN excels at capturing fine-grained spatial patterns and domain boundaries through graph convolutional networks, although it requires careful parameter tuning. The optimal approach depends on data resolution: BinSpect combined with BayesSpace performs best for low-resolution data with gradual transitions, while MERINGUE or SpatialDE paired with Louvain clustering are preferable for high-resolution data with distinct boundaries. SPARK provides a robust default choice balancing sensitivity and specificity across diverse applications [116,117].

Spatial information can also be used for cellular communication analysis. For tumor studies, ligand–receptor (L–R) pairing and co-localization, which cannot be decoded by a single cell, may be more responsible for heterogeneity. AI-powered methods have revolutionized this domain: Gene Chart Convolutional Neural Network (GCNG), DeepTalk, CellChat-V2, and CellPhoneDB v3.0 for analyzing cell-to-cell gene interactions [118–120]; SVCA for quantifying cell-to-cell interactions [121]; MISTy using a multi-slice approach for simulating cell interactions [110]; and NICHES for visualizing L–R expression profiles [122]. Some researchers have established SpaTalk by modelling and scoring the L–R signaling network based on mapping, non-negative linear regression, and ML [123]. The COMMOT method infers cell–cell communication in spatial transcriptomics via collective optimal transport [124].

### Prospects for overcoming the heterogeneous data challenge

The integration of multimodal spatial omics data represents one of the most challenging yet promising frontiers, where AI and deep learning methods are proving indispensable. The number and level of exploration of existing technologies make the heterogeneous data challenge difficult to solve, mainly in the context of methodological applications that are mostly confined to research centers and not available for widespread use. Commonly used databases, such as SpatialDB and STOmicsDB, provide interactive visualization platforms but lack built-in spatial annotations. Platforms such as STOmics and CROST mainly summarize sequencing correspondence data. Users are not allowed to upload their own data because existing platforms rely on heterogeneous pipelines to handle different data formats [125,126].

Research is now making significant breakthroughs in cross-modal data fusion [87,127]. Deep learning frameworks capable of multimodal integration can now combine spatial transcriptomics with proteomics, metabolomics, and imaging data, learning shared latent representations across modalities. Variational autoencoders (VAEs), generative adversarial networks (GANs), and attention-based transformer architectures are being adapted to align and integrate heterogeneous spatial omics datasets, addressing challenges in batch effect removal, domain adaptation, and missing data imputation [128,129]. These AI algorithms show tremendous potential to enable large-scale, standardized use of spatial omics methods across research centers, revolutionizing our ability to construct comprehensive, multimodal molecular atlases of tissues in health and disease. A critical consideration in successful implementation is scalability and generalizability across different data types and experimental conditions [92,130]. Recent systematic evaluations reveal that many computational methods, particularly graph neural network-based approaches, perform optimally with default settings on specific data types but require parameter customization for others, highlighting opportunities for refinement through careful tuning of input features and clustering algorithms. These findings underscore the need for flexible analytical frameworks that accommodate the diverse characteristics of spatial omics data across different technologies and biological contexts. As computational power increases and training datasets grow, we anticipate that AI-driven spatial omics analysis will become increasingly accessible, accurate, and biologically meaningful, bridging the gap between technological capability and widespread clinical and research application.

### Application of spatial omics in lung cancer

**Figure 4:**
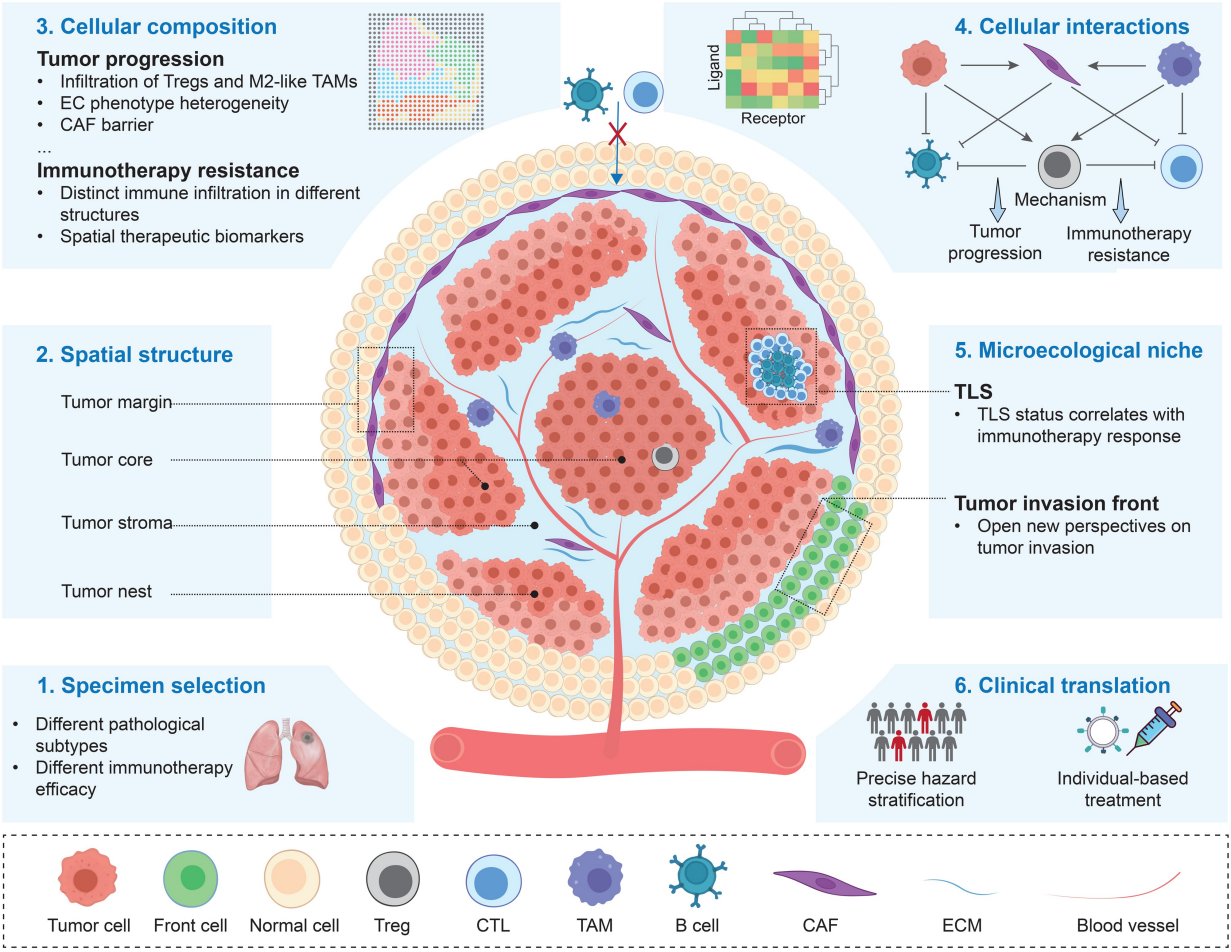
Feasible strategies for the application of spatial omics in lung cancer research By collecting tumor tissue samples from different patients and performing spatial structure partitioning, we can explore the spatial heterogeneity of lung cancer and further analyze tumor progression and immune resistance mechanisms for precise prognostic stratification and personalized treatment. TLS, tertiary lymphoid structure; TAM, tumor-associated macrophage; Treg, regulatory T cell; CAF, cancer-associated fibroblast; CTL, cytotoxic T cell; ECM, extracellular matrix.

With the continuous advancement of spatial omics technologies, numerous studies in recent years have explored lung cancer heterogeneity from the perspective of spatial ecosystems. These studies have achieved significant progress in elucidating mechanisms of lung cancer progression and drug resistance, developing novel prognostic or therapeutic biomarkers, and identifying new therapeutic targets (**Table 3**). This section proposes relevant strategies for applying spatial omics technologies in lung cancer research (**Figure 4**) and provides potential directions for future investigations. Another major use of ST is in developmental evolution studies, although the focus of this paper is on tumors, and this topic will not be expanded upon here.

**Table 3:** Advancements of spatial omics in lung cancer research

Study	Human specimen	Method	Division of spatial structure	Application	Cellular composition and distribution	Intercellular interaction	Cellular neighbourhood	Feasibility of clinical translation	Ref.
Larroquette M et al., 2022	NSCLC	GeoMX, IHC, scRNA-seq, bulk RNA-seq	TN & TS	(1) Mechanisms of resistance to ICI (2) Markers of ICI responsiveness	Patients with low TAM infiltration have a good response to ICI, while TAM level in TS is not related to the response to ICI	–	–	The high expression of CD27, CCL5, and ITGAM in TN is related to TAM recruitment, which may represent potential therapeutic targets	[131]
Moutafi MK et al., 2022	Advanced NSCLC	GeoMx, IF	TN & TS	(1) Mechanisms of resistance to ICI (2) Markers of ICI responsiveness	CD44 expression is higher in TS than in TN	–	–	For patients receiving ICI, CD44 expression in TN, but not TS, is strongly associated with longer PFS	[132]
Song X et al., 2023	Advanced NSCLC	GeoMx, IHC	TN & TS	(1) Mechanisms of resistance to ICI (2) Markers of ICI responsiveness	Immune infiltration is significantly higher in TS than in TN	–	–	Markers from TS have better predictive ability for immunotherapy response	[133]
Monkman J et al., 2023	Advanced NSCLC	GeoMX, IHC	TN & TS	(1) Mechanisms of resistance to ICI (2) Markers of ICI responsiveness	In ICI-responsive patients, TN shows high IL-2R and low CD44 expression, while TS exhibits low CTLA-4, IDO-1, SPP1, and CD56 expression	The interactions between TAMs and PD1^+^/FoxP3^+^ cells are enriched in ICI refractory tumors	–	A prediction model of ICI response is established based on IL-2, CD44, SPP1 and other indicators	[134]
Yan Y et al., 2024	Resectable NSCLC	GeoMx, 10x Visium, scRNA-seq, bulk RNA-seq	TN & TS Core & Margin	(1) Mechanisms of resistance to ICI (2) Markers of ICI responsiveness	Both SPP1^+^ TAMs and COL11A1^+^ CAFs are enriched at the tumor margin and form a barrier surrounding TCs	(1) CAFs interact with TCs via DDR1-COL11A1 pathway, promoting the formation of a collagen fiber barrier (2) TAMs interact with CAFs via SPP1-CD44 pathway to suppress T cell infiltration	Activated TLS exerts anti-tumor effects, while late TLS reflects the decline of the immune response	COL11A1^+^ CAFs and SPP1^+^ TAMs are associated with poorer responses to immunotherapy, while activated TLS is linked to better immunotherapy responsiveness	[135]
Cole M et al., 2024	KRAS^+^ NSCLC	IMC	Core & Margin	(1) Mechanisms of resistance to ICI (2) Markers of ICI responsiveness	T and B cells are excluded from the tumor region, whereas KRAS-G12C inhibitor treatment induced infiltration of T cells and antigen-presenting cells	CXCL9-high DCs drive anti-tumor immunity via the recruitment of activated T cells	The T/DC community may provide an activatory niche for anti-tumor cytotoxic T cells, a function likely suppressed by the presence of Tregs	Depleting Tregs may enhance tumor control by combining anti-PD-1 therapy with KRAS-G12C inhibitors, potentially improving patient OS	[136]
Chen H et al., 2025	SCLC	CODEX, CosMx, WES, scRNA-seq	Core & Margin	(1) Mechanisms of resistance to ICI (2) Markers of ICI responsiveness	Over 10% of tumor cells co-express ASCL1/NEUROD1, particularly enriched in the ASCL1 subtype (SCLC-A)	ASCL1^+^ TCs are located adjacent to vasculature and exhibit robust tumor–vascular interactions	MPTC-enriched CN9 confers an aggressive phenotype via high SLFN11/RBBP6 mutations	The MT2 niche, composed of M1-TAMs, CD8^+^ T cells, and NKT cells, serves as a predictor for immunotherapy response	[137]
Aung TN et al., 2025	Advanced NSCLC	CODEX, GeoMx	TN & TS	(1) Mechanisms of resistance to ICI (2) Markers of ICI responsiveness	TCs are most abundant in the TN region, whereas the TS region is predominated by M1/M2-TAMs and CAFs	Non-progressors exhibit stronger interactions between M2-TAMs and CD4^+^ T cells, as well as between M2-TAMs and CD8^+^ T cells	In patients with disease progression, TCs, Neus, and vasculature co-aggregate into CNs that promote tumor proliferation and angiogenesis	An immune resistance signature and a response signature are identified in NSCLC, demonstrating robust predictive performance for immunotherapy response	[138]
Wu F et al., 2023	SCLC with high ASCL1 expression	scRNA-seq, 10x Visium, mIF, IHC	Core & Margin	(1) Mechanisms of tumor invasion (2) Prognostic markers	ASCL1 is highly expressed in the tumor core, while tumor-derived Ig is highly expressed in the tumor margin and shows invasive features	SLAMF7 signaling between tumor cells is highly expressed and further activated at the invasive front		ASCL1^–^lg^+^ patients have worse PFS and OS than ASCL1^+^ lg^–^ patients	[139]
Sorin M et al., 2023	LUAD	IMC	Histological subtypes	(1) Mechanisms of subtype progression (2) Prognostic markers	The degree of immune infiltration and M2-like TAMs are the highest in solid type	(1) TCs tend to have homotypic interactions (2) As the subtype progresses, the interaction of TAMs with T, B, and ECs increases	Ten kinds of CNs are defined	CD4^+^ T cells and B cell-enriched CNs are linked to better survival, while Ki-67^+^ ECs and HIF1α^+^ Neus are associated with worse prognosis	[140]
Peng H et al., 2023	IA-IIIB NSCLC	mIF, scRNA‐seq	TN & TS	(1) Mechanisms of tumor progression (2) Prognostic markers	CD38^+^ T cells and M2-like TAMs are enriched in TN, while CSCs, CD4^+^ T cells and CD20^+^ B cells are enriched in TS	Spatial proximity of T or B cells to N1-like Neus promotes anti-tumor immunity, while Tregs near Neus reduce immune infiltration	The proximity among CD4^+^ T cells, CD20^+^ B cells, and CD38^+^ T cells contributes to the formation of TLS	(1) The spatial proximity between T/B cells and N1-like Neus is associated with better prognosis (2) Spatial proximity between CSCs is associated with worse prognosis	[141]
Wang Y et al., 2023	LUAD	10x Visium, IHC	Histological subtypes	Mechanisms of subtype progression	TAM phenotypic heterogeneity, hypoxia, immunosuppressive TME, and dedifferentiation are the key features of LUAD subtype progression	MIF-TNFRSF14 signaling is highly expressed in aggressive LUAD subtypes	–	–	[142]
Takano Y et al., 2024	LUAD	10x Visium, Xenium, CODEX	Low malignant zone & high malignant zone	Mechanisms of subtype progression	With tumor progression, EGFR^+^ LUAD TCs are more malignant; KRAS^+^ LUAD TCs can also highly express mucin to protect themselves and wrap CAF to avoid immune attack	As tumor progresses, the interaction between SPP1^+^ TAMs and CAFs is enhanced in EGFR^+^ LUAD to promote EMT, while KRAS^+^ LUAD TCs activate IDO1 to inhibit T and NK cell activity	–	Even within the immunosuppressive microenvironment, a subset of CD8^+^ T cells retain cytotoxic activity, suggesting that ICI may still exhibit therapeutic efficacy	[143]
Enfield KSS et al., 2024	NSCLC	IMC, IHC, WES, bulk RNA-seq	TN & TS Core & Margin	(1) Mechanisms of tumor invasion (2) Prognostic markers	Fibroblasts are distributed at the tumor margin, possibly inhibiting immune infiltration	In the TME with high Neus infiltration, the interaction between Neus and cytotoxic T cells is low	High clonal neoantigen burden is linked to increased T and MΦ-enriched CNs in LUAD, and B- and plasmocyte-enriched CNs in LUSC	High Neus infiltration is associated with shorter DFS	[144]
Cords L et al., 2024	NSCLC	IMC	TN & TS	(1) Mechanisms of tumor progression (2) Prognostic markers	(1) 11 types of CAF are defined (2) MA CAFs, mCAFs, and ifnCAFs accumulate in TS, whereas hypoxic tCAFs and iCAFs accumulate in TN	–	vCAF- and iCAF-enriched CNs are adjacent to vascular and lymphatic ECs	ifnCAF, iCAF, and SMA CAF are associated with longer survival, wheras tCAF, hypoxic tCAF, collagenCAF, and mCAF are associated with shorter survival	[145]
Xie L et al., 2024	LUAD	10x Visium, GeoMx, scRNA-seq	Lepidic & Acinar	Mechanisms of subtype progression	Lepidic ECs can regulate angiogenesis and promote the activation and migration of immune cells, whereas acinar ECs promote apoptosis	Compared with lepidic, acinar ECs inhibits CD8^+^ T cell infiltration by high expression of PD-L1	–	–	[146]

*Note*: NSCLC, non-small cell lung cancer; TAM, tumor-associated macrophage; ICI, Immune checkpoint inhibitor; TC, tumor cell; Neu, neutrophil; CN, cellular neighborhood; CSC, cancer stem cell; EC, endothelial cell; TN, tumor nest; TS, tumor stroma; LUAD, lung adenocarcinoma; OS, overall survival.

### Spatial structural division of tumor tissues

Tumors are complex spatial ecosystems with intricate structural organization. While scRNA-seq loses the spatial context of individual cells, spatial omics technologies can precisely reveal the anatomical structure of samples, facilitating the analysis of heterogeneity across different tumor tissue architectures. In lung cancer research, tumors are often divided into tumor nests and stroma, or core and margin regions. Analyzing the immune infiltration heterogeneity between these structures provides valuable insights into tumor invasion and mechanisms of resistance to immunotherapy. Moreover, a distinctive feature of lung adenocarcinomas is the coexistence of multiple histological subtypes within the same tumor tissue [147,148]. These subtypes exhibit varying degrees of invasiveness and malignancy. Spatial omics enables accurate delineation of these subtypes within samples, allowing for the analysis of their heterogeneity and exploration of mechanisms driving subtype progression.

### Cellular composition and distribution

scRNA-seq has emerged as a pivotal tool for deconstructing the TME, constructing cellular atlases, and identifying functional cell subpopulations. For instance, studies using scRNA-seq have identified distinct cellular subsets — such as TM4SF1^+^/SCGB3A2^+^ malignant cell subpopulations, FAP^+^/PDPN^+^/α-SMA^+^ cancer-associated fibroblasts (CAFs), and GZMK^+^ CD8^+^ T cells — which have been demonstrated to be closely associated with key pathological processes including tumor progression and immune evasion [149–151]. However, despite its high resolution in delineating cellular identities and transcriptomic profiles, scRNA-seq does not preserve the native spatial context of cells within intact tissue. This limitation hinders the ability to uncover the spatial distribution patterns of these cell subsets throughout the tumor mass. In contrast, spatial omics technologies enable systematic investigation of heterogeneity in cellular composition and gene expression across distinct tumor regions, thereby advancing our understanding of tumor biology in the spatial dimension. For instance, the degree of immune infiltration tends to increase during the progression of lung adenocarcinoma subtypes [140,152]. However, in high-grade solid subtypes, an immune exclusion phenotype is observed, with immune cells predominantly accumulating at the tumor margin [142,152]. Studies have shown that this immune exclusion phenotype is driven by the infiltration of regulatory T cells (Tregs), M2-like tumor-associated macrophages (TAMs), coupled with the formation of a physical immune barrier by CAFs [135,142–145,153–155]. Endothelial cell (EC) phenotype heterogeneity also contributes to the progression of adenocarcinoma subtypes. In low-grade subtypes, ECs promote immune activation, while in high-grade subtypes, ECs facilitate cell apoptosis [146]. In immunotherapy, studies have found that the tumor nest and stroma exhibit distinct immune cell characteristics, and both have different predictive abilities for treatment response (Table 2) [131–134,144]. It is worth noting that Moutafi MK et al. found that CD44 expression in the tumor nest enhanced the efficacy of immunotherapy [132], while Monkman J et al. reached the opposite conclusion [134]. Therefore, further exploration is needed. In addition, even within the immunosuppressive microenvironment, a subset of CD8^+^ T cells retains cytotoxic activity, suggesting that immune checkpoint inhibitors (ICIs) may still exhibit therapeutic efficacy [143]. These findings may pave the way for the development of next-generation immunotherapies.

### Cellular interactions

Cell–cell interactions within the tumor ecosystem contribute to our understanding of the mechanisms underlying the formation of the specific cellular distributions described above. Notably, while scRNA-seq can be used to infer cellular interactions, its analyses remain inherently speculative — being derived indirectly from transcriptomic data lacking preservation of cells’ native spatial contexts. In contrast, spatial omics technologies enable direct visualization of spatially proximal ligand-receptor pairs within the tissue microenvironment, thereby providing tangible and topologically grounded evidence for cellular interactions and significantly strengthening the biological validity of cell communication studies. Overall, in lung cancer, tumor cells tend to engage in homotypic interactions, suggesting a potential strategy to evade immune system attacks [140]. In heterotypic interactions, tumor cells can evade immune cell attack and enhance their invasion capacity by expressing various markers, such as IDO1, TGF-β, MIF, and NRF2 [135,142,143,154]. Tumor cells can also interact with CAFs through DDR1 to promote the formation of a collagen fiber barrier and indirectly inhibit the penetration of immune cells [135]. In addition to tumor cells, interactions among other cell types within the TME also play a crucial role. The interactions between TAMs and T cells, B cells, or ECs are enhanced, thereby exerting immunosuppressive effects [140,141,143]. CAFs can interact with SPP1^+^ TAMs through SPP1-CD44 signaling to promote the proliferation and activation of each other and inhibit the infiltration of T cells [135,143]. These interactions not only reveal the mechanism of tumor progression but also suggest the cause of immune resistance, helping us better understand the TME.

### Tumor microecological niche

The cell–cell interaction analyses mentioned above are mostly limited to interactions between two cell types, but tumors, as complex organisms, often rely on the coordinated action of multiple cell types to accomplish certain functions. We can refer to this multicellular functional structure as the tumor microecological niche. While scRNA-seq fails to preserve spatial context, its analysis is largely confined to the single-cell level, thereby limiting insights into the multicellular interplay that defines tumor microecological niches. In contrast, spatial omics offers distinct advantages in resolving such tissue-level architecture. One widely studied tumor microecological niche is the tertiary lymphoid structure (TLS), which consists of a central region rich in B cells, surrounded by a peripheral area populated by T cells, dendritic cells (DCs), and macrophages [156,157]. In lung cancer, interactions among CD20^+^ B cells, CD4^+^ T cells, and CD38^+^ T cells have been shown to play a critical role in the formation of TLS [141]. During lung cancer progression, TLS initiation begins with the aggregation of CD4^+^ T cells and CXCL13^+^ T follicular helper cells, followed by the accumulation of B cells, ultimately forming aggregates of CD4^+^ T cells and B cells [158]. Yan et al. divided TLS into four categories: early TLS, activated TLS, declining TLS, and late TLS. They found that activated TLS had anti-tumor effects, while late TLS reflected a decline in the immune response [135]. Wang et al. discovered that type II alveolar epithelial cell (AT2) surrounding TLS suppresses B cells within TLS via the MIF-SIAE signaling axis, thereby exerting an immunosuppressive effect [159]. Notably, in recent years, the tumor invasion front has become a focal point for exploration [160]. In small-cell lung cancer (SCLC), tumor-derived immunoglobulins are enriched at the invasion front, exhibiting invasive characteristics [139]. In NSCLC, studies have identified more active oncogenic signaling pathways in the peripheral cancer regions, but no specific analysis of the invasion front has been conducted [154]. Future research focusing on the invasion front is expected to further clarify the mechanisms of invasion and metastasis in lung cancer.

### Feasibility of clinical translation

The preceding sections have systematically elucidated the value of spatial omics in studying lung cancer progression and immune resistance. To better translate these cutting-edge findings into clinical practice, recent research has begun to correlate them with survival outcomes such as disease-free survival and overall survival (OS), aiming to identify novel biomarkers. For instance, Cole et al. employed IMC to identify an immunologically active niche enriched with CD4^+^ T cells, CD8^+^ T cells, and dendritic cells in *KRAS*-mutant lung cancer, which was frequently suppressed by Tregs. Depleting Tregs may enhance tumor control by combining anti-PD-1 therapy with KRAS-G12C inhibitors, potentially improving patient OS [136]. Using mIF and CosMx, Chen et al. identified an MT2 immune niche — composed of M1-like macrophages, CD8^+^ T cells, and natural killer T cells — that was significantly associated with prolonged OS, suggesting improved response to immunotherapy [137]. Furthermore, advances in ML algorithms now enable the integration of multiple spatial features to develop more accurate prognostic models [161]. Aung et al. utilized CODEX and GeoMx to identify immune resistance signatures and immune response signatures in NSCLC, training ML models that demonstrated robust performance in predicting immunotherapy response across multiple external validation cohorts [138]. Zhang et al. applied GeoMx to assess intra-tumor heterogeneity in SCLC in three dimensions, developing the ITHtyper model, which achieved effective prognostic stratification in multicenter validation cohorts [162]. Meng et al. employed mIHC to identify a T cell-enriched cellular neighborhood (CN) and an immune-suppressed-enriched CN associated with lymph node metastasis [155]. By further integrating NGS-derived features, they developed an ImGene model, which demonstrated robust performance in predicting lymph node metastasis in lung adenocarcinoma. Building on these findings, we anticipate that emerging technologies such as cancer vaccines may eventually target the newly discovered spatial biomarkers, ultimately transforming the landscape of cancer treatment [163,164].

## Challenges and the prospects of spatial omics

**Figure 5:**
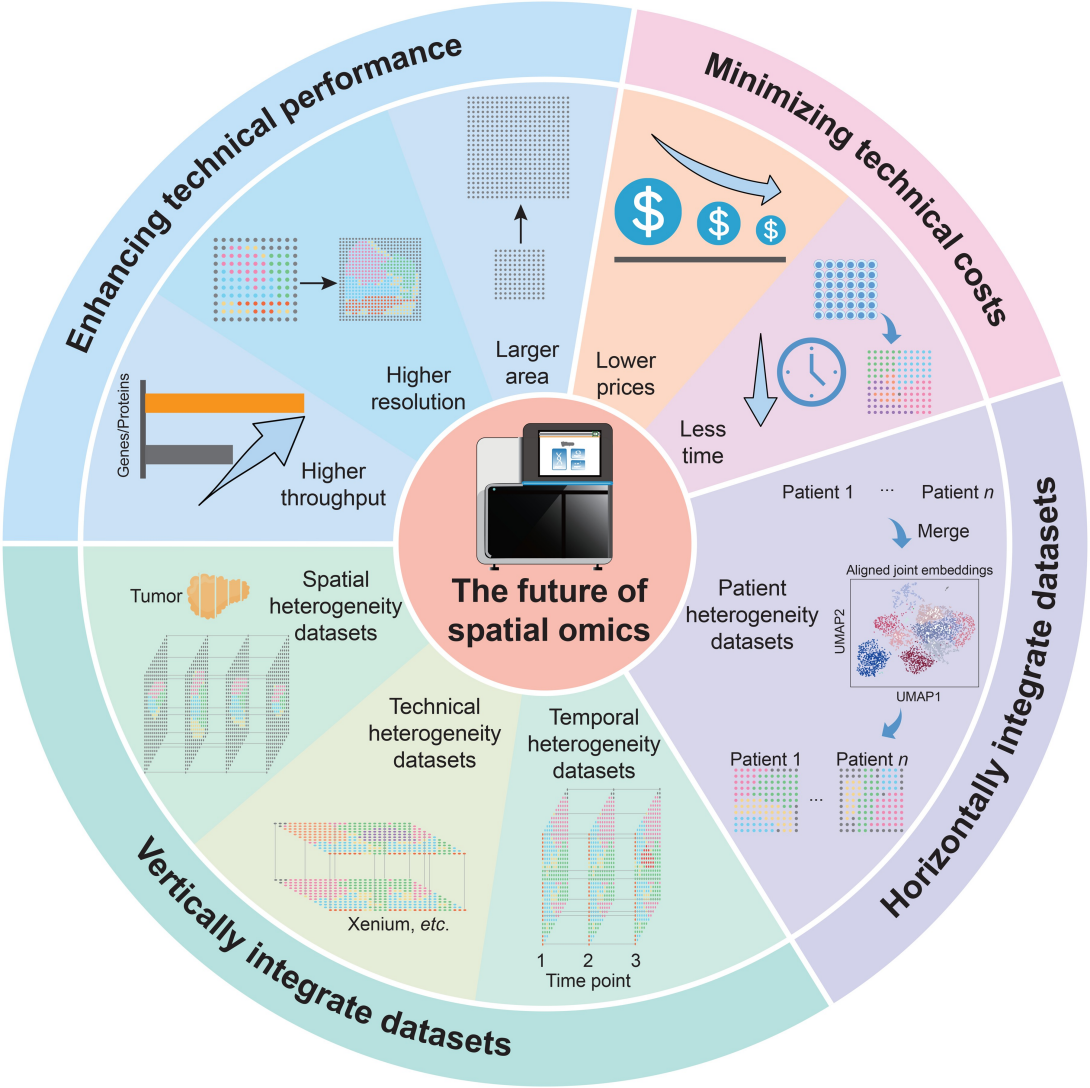
Future directions of spatial omics Advances in technologies help us create more precise spatial omics maps at lower costs, while improvements in algorithms enable the integration of heterogeneous data for multimodal analysis, thereby uncovering more insights.

Lung cancer remains the leading cause of cancer-related deaths. The existence of tumor heterogeneity results in significant differences in treatment sensitivity and prognosis among patients. The rapid development of spatial omics technologies has provided new insights into tumor heterogeneity. In practice, the high-throughput advantages of sequencing-based ST technologies can first be leveraged to initially identify potential biomarkers. Then, the spatial characteristics of these biomarkers of interest can be further investigated using SP or imaging-based ST technologies with ultra-high resolution. Building upon this foundation, the role of key biomarkers in lung cancer progression and immune resistance can be explored and integrated to elucidate the underlying mechanistic pathways, and ultimately enable precise patient stratification and personalized treatment strategies. To achieve these goals, current spatial omics platforms still have substantial room for improvement: (1) Enhance throughput and resolution for accurately mapping spatial omics profiles while maintaining high sensitivity. Emerging technologies such as HDST and advanced multiplexed imaging platforms have demonstrated the feasibility of achieving subcellular resolution with thousands of targets. Optimizing probe design strategies and implementing AI-driven image analysis algorithms can further improve both sensitivity and resolution while reducing background noise. (2) Reduce technical and time costs to make practical implementation feasible. The development of microfluidic-based automation systems and streamlined library preparation protocols can significantly decrease experimental time from weeks to days. Open-source computational pipelines and cloud-based analysis platforms can lower the barriers to data processing. Furthermore, cost-sharing through multi-center consortia and the establishment of core facilities can make these technologies more accessible in clinical research settings. (3) Align heterogeneous data across spatiotemporal platforms, detection technologies, and omics domains. This challenge encompasses integrating data from different spatial omics platforms, detection methodologies, and biological layers. Two critical integration paradigms require particular attention: spatial integration addresses spatial heterogeneity by combining data from different sampling locations (*e*.*g*., distinct tumor regions, multiple biopsies in the same region, or different anatomical sites), capturing how spatial microenvironments generate distinct molecular profiles; temporal integration addresses temporal heterogeneity by integrating data across disease timelines (*e*.*g*., pre-treatment biopsies, surgical specimens, and post-relapse samples), revealing tumor evolution and treatment responses over time. Standardized data formats and interoperable frameworks (*e*.*g*., the SpatialData ecosystem) can facilitate cross-platform integration. ML approaches, including transfer learning and domain adaptation algorithms, show promise in harmonizing multi-platform data. Graph-based methods and multi-view learning can effectively integrate spatiotemporal dynamics and multi-omics layers. Establishing common coordinate systems, reference atlases, and spatial-specific batch correction algorithms will be essential for robust data alignment. (4) Promote the consistency and scalability of benchmarks and metrics across spatial datasets. The spatial omics community should establish standardized quality control metrics and reference datasets for method validation. Initiatives such as ST benchmarking consortia can provide gold-standard datasets and evaluation frameworks. Implementing FAIR (Findable, Accessible, Interoperable, Reusable) data principles and creating centralized data repositories will enhance reproducibility and enable cross-study comparisons (**Figure 5**). In conclusion, we anticipate that spatial omics technologies will provide new perspectives and methods for lung cancer research and open new avenues for precision medicine.

## CRediT author statement

**Yue He:** Conceptualization, Methodology, Investigation, Data curation, Writing – original draft, Visualization. **Zifan Li:** Methodology, Investigation, Validation, Writing – original draft, Visualization. **Wenxiang Wang:** Investigation, Writing – review & editing. **Xu Liu:** Investigation, Writing – review & editing. **Shanshan Lu:** Supervision, Writing – review & editing. **Jing Bai:** Visualization, Writing – review & editing. **Lin Weng:** Visualization, Writing – review & editing. **Qingna Zhang:** Project administration, Writing – review & editing. **Jun Wang:** Project administration, Investigation, Supervision, Funding acquisition, Writing – review & editing. **Kezhong Chen:** Conceptualization, Supervision, Writing – review & editing, Funding acquisition, Project administration. All authors have read and approved the final manuscript.
